# Gut Microbiota Dysbiosis Facilitates Susceptibility to Bloodstream Infection

**DOI:** 10.1007/s12275-024-00190-5

**Published:** 2024-12-02

**Authors:** Xiaomin Lin, Chun Lin, Xin Li, Fen Yao, Xiaoling Guo, Meimei Wang, Mi Zeng, Yumeng Yuan, Qingdong Xie, Xudong Huang, Xiaoyang Jiao

**Affiliations:** 1 Department of Clinical Laboratory, Jieyang People’s Hospital, Jieyang, 522000 Guangdong People’s Republic of China; 2 Department of Clinical Laboratory, The First Affiliated Hospital of Shantou University Medical College, Shantou, 515041 People’s Republic of China; 3 Department of Cell Biology and Genetics, Shantou University Medical College, Shantou, 515041 Guangdong People’s Republic of China; 4 Department of Pharmacology, Shantou University Medical College, Shantou, 515041 Guangdong People’s Republic of China

**Keywords:** Bloodstream infection, Intestinal flora, 16S rRNA gene sequencing, Pathogen

## Abstract

To study the role of intestinal flora in the development of bloodstream infections (BSIs). 42 patients and 19 healthy controls (HCs) were screened into the study and their intestinal flora was measured by 16S rRNA gene sequencing. The bacterial diversity was significantly lower in the BSI group compared with that in the HCs (P < 0.001), and beta diversity was significantly differentiated between the two groups (PERMANOVA, P = 0.001). The four keystone species [*Roseburia*, *Faecalibacterium*, *Prevotella*, and *Enterococcus* (LDA > 4)] differed significantly between the two groups. Dysbiosis of fecal microbial ecology is a common condition present in patients with BSI. The proliferation of certain pathogens or reduction of SCFA-producing bacteria would cause susceptibility to BSI.

**Supplementary Information:**

The online version contains supplementary material available at 10.1007/s12275-024-00190-5.

## Introduction

Around the world, bloodstream infections (BSIs) occur frequently and can lead to serious morbidity and mortality, e.g., sepsis (Kontula et al., 2021). The pathogens for BSI can originate from external places (external environment) and internal places (gut, and skin) (Tamburini et al., 2018). The human gastrointestinal tract contains trillions of bacteria that compose an intricate ecosystem, and some of them can be opportunistic pathogens. However, the intestinal barrier can separate the intestinal ecosystem from the host, blocking gut bacteria from entering the bloodstream (Kim et al., 2017). In addition, intestinal pathogens are inhibited by resident microbiota via tuning on the host immune response and can be outcompeted for resources such as nutrition, metabolites, and space. However, infection often begins with perturbation of the ecosystem (Buffie & Pamer, 2013). An example is the thinning and damage to the intestinal barrier in certain patients (Rupani et al., 2007). The compromised barrier would allow pathogens to move from the gut into circulation to cause primary bloodstream infections (See et al., 2017). Thus, most sepsis-causing bacteria originated from the gut which also exhibited altered baseline microbiota composition among these patients (Bassetti et al., 2020). Among such pathogens, dominance by *enterococcus* was associated with a nine-fold increase of subsequent vancomycin-resistant Enterococcal bacteremia, whereas proteobacterial dominance with a five-fold increase of gram-negative bacteremia (Miller et al., 2021). Furthermore, bacteremia developed after these pathogens had control of the digestive tract for an average of 7 days (Taur et al., 2012), and changes in microbiota compositions influenced the subsequent development and severity of BSI (Bassetti et al., 2020).

The intestinal microbiota of individuals with immunological dysregulation is involved in the onset, progression, and prognosis of sepsis (Miller et al., 2021). Furthermore, certain changes in intestinal flora were used to anticipate the start of illness before symptoms manifest (Gilbert et al., 2016) and were used in clinical trials (Dickson, 2016). Among different intestinal microbiota-associated diseases, the pathogenesis of sepsis is highly intricate, in part because of biological redundancy prevailing in the microbiota (Schuijt et al., 2013). It has been stated that there may be an "incompletely known bidirectional link" between BSI/sepsis and the microbiome (Bassetti et al., 2020), however, there has been limited data on how dysbiosis affected BSI in clinical practice, thus, extensive data of the role of the intestinal flora in the BSI are urgently needed (Bassetti et al., 2020; Miller et al., 2021). A major aim of our study was to evaluate the compositions of gut microbiota in BSI patients, discovering significant bacteria or bacterial profiles that play a main role in BSI pathogenesis.

## Materials and Methods

### Ethics and Study Design

This retrospective cohort study was conducted at Jieyang People's Hospital from June to October 2021. The study was approved by the Institutional Ethics Board of Jieyang People’s Hospital, including all procedures (NO. 2022028). The fecal specimens were left over from previous clinical diagnoses, and the clinical data were known by checking the case review. All data are anonymous, so the patients were exempted from informed consent according to the approval of the hospital's ethical committee.

### Participants

Patient inclusion criteria: All participants were older than 18 years. BSI was designated when live bacteria were detected in blood samples from patients. BSI will not be diagnosed if the isolates are the skin contaminant or colonizing bacteria (e.g., *coagulase-negative Staphylococci*), unless the same bacteria was cultured from the blood samples taken from two different sites, or the bacteria isolated from the blood was the same as isolates from other body sites.

Exclusion criteria included: (1) Blood cultures with suspected contaminating bacteria; (2) Incomplete medical records; (3) No routine stool specimens before or within four days after the blood culture. Healthy controls (HCs) were healthy individuals who had no infections and did not use antibiotics or probiotics within the previous three months. In addition, their routine physical, biochemical, and imaging examinations were in the normal ranges. All participants were Chaoshanese living in Jieyang with similar eating habits. Their gender, age, comorbidities, blood and biochemical biomarkers, and pertinent clinical microbiology results were recorded.

### Bacterial Identification and Blood Parameters Measurement

The BacT/ALERT® 3D™ system (Becton–Dickinson, USA) was used to culture blood samples. The incubation was carried out for a maximum of 5 days. Species were identified by a VITEK II (bioMérieux, France). The blood routine parameters were measured by an automated system (Sysmex XN-2000). Procalcitonin (PCT) was measured with the electrochemiluminescence method using an automated system (Roche Cobase 411). The biochemical parameters were measured by a Hitachi 7600 Series Automatic Biochemical Analyzer.

### Fecal Sample Collection

During the study period, all fecal specimens from the Laboratory Department that had done routine fecal testing programs were collected and stored at – 80 °C until the blood cultures of the corresponding patients were positive. These specimens were then used for DNA extraction. Specimens from patients with negative blood cultures were excluded.

### Intestinal Flora Measurement

Total genomic DNA was extracted from the stool samples (Lutz et al., 2011), and its quality was analyzed using 1% agarose gel electrophoresis. The distinct regions (16S V3-V4) of genes were amplified by PCR (Phusion® High-Fidelity PCR Master Mix, New England Biolabs), with primers 341F (5’-CCTAYGGGRBGCASCAG-3’) and 806R (5’-GGACTACNNGGGTATCTAAT-3’) with the barcode. 2 µM of forward and reverse primers, and about 10 ng of template DNA. Thermal cycling consisted of initial denaturation at 98 °C for 1 min, followed by 30 cycles (98 °C, 10 s; 50 °C, 30 s; 72 °C, 30 s), then a final extension step at 72 °C for 5 min. The libraries were generated using the TruSeq® DNA PCR-Free Sample Preparation Kit (Illumina, USA). The library’s quality was assessed on the Qubit@ 2.0 Fluorometer (Thermo Scientific). On the Illumina NovaSeq platform, the library was finally sequenced, and 250 bp paired-end reads were produced.

### Bioinformatics Analyses

For subsequent bioinformatics analysis, demultiplexed sequences from each sample were quality-filtered and trimmed, de-noised, and merged. The chimeric sequences were identified and removed using the QIIME2 dada2 plugin (Caporaso et al., 2010). Using UPARSE version 7.0.1001, filtered sequences were grouped into operational taxonomic units (OTUs) based on 97% identity (Edgar, 2013). By contrasting them with the Greengenes reference database 13_8, taxonomic ranks were given to OTU representative sequences. Alpha and beta diversity analyses were performed using QIIME2, based on a normalized OTU table. We performed principal coordinate analysis (PCoA) analysis based on the Bray Curtis distance and selected the combination of principal coordinates with the largest contribution for graphical presentation. The results were visualized using the Phyloseq package in R. The analysis of partial least squares discriminant analysis (PLS-DA) was done by the mixOmics package in R. PLS-DA is a supervised analysis method, which needs to group the test samples according to the categories, and differentiate the groups when calculating the mathematical model, ignoring the random differences within the groups and highlighting the systematic differences between the groups, but it may be subject to the phenomenon of overfitting. To determine the impact of each microbiota taxon's abundance on the effect of difference and to pinpoint microbiota taxa with significant differences in their demarcation, the line discriminant analysis (LDA) of effect size (LEfSe) was utilized (Segata et al., 2011).

### Statistical Analyses

Compositional differences between the two groups were analyzed by descriptive statistics, while categorical and continuous variables were analyzed by the chi-square test, the two-sample t-test, or the Mann–Whitney U-test. Sequencing data were analyzed using R software (v4.0.2) and SPSS 26.0 software, and p-values represented two-sided statistical tests. Statistical significance was defined as a value of P < 0.05. To track changes in microbial communities linked to variables stored as metadata, permutational multivariate analysis of variance (PERMANOVA) was used. Relationships between clinical factors and bacterial species were examined using Spearman’s correlation analysis, and heatmaps were drawn using the 'heatmap' package in R software (v4.0.2). The relative abundance of enteric flora bacteria with LDA values greater than 4 at the genus level was used to construct the corresponding receiver operating characteristic curves (ROC), respectively. Multi-indicator co-diagnosis was performed by using the relative abundance of gut flora bacteria with LDA values greater than 4 at the genus level as the independent variable and grouping as the dependent variable, fitting a binary logistic regression model using SPSS 26.0 software, calculating the predicted probability, and then using the predicted probability as the test variable for the ROC curves, and grouping as the state variable, and then plotting the ROC curves using GraphPad Prism 9.0.

## Results

### Clinical Features and Biochemical Parameters

42 BSI patients were recruited according to our inclusion and exclusion criteria. The BSI group included 17 males and 25 females, with an average age of 64.64 ± 11.08 years. 19 HCs (8 males and 11 females, with an average age of 63.95 ± 8.42) were recruited. There was no significant difference according to age and gender in the two groups. For blood parameters, white blood cells (WBC), neutrophils, aspartate aminotransferase (AST), and uric acid (UA) levels were higher in the BSI group than those in the HCs group (P < 0.05). C-reactive protein (CRP) and PCT were significantly higher than the normal range in the BSI group, indicative of the infection status of the patients. In addition, most patients had some underlying diseases, the most common being diabetes (42.86%). The most common pathogen isolated from the patients was *Escherichia coli* (Table 1).

**Table 1:** Clinical and demographic information of patients

	BSI (n = 42)	HCs (n = 19)	Normal range	P-Value
Demographics				
Age (years)	64.64 ± 11.08	63.95 ± 8.42	N/A	0.809
Gender (%male)	17 (40.48%)	8 (42.1%)	N/A	0.905
Laboratory parameters				
WBC (× 10^^9^/L)	12.08 (7.46–17.06)	7.09 (5.46–8.42)	3.50–9.50	0.001
NEUT (× 10^^9^/L)	10.15 (6.57–15.10)	3.83 (2.90–4.66)	1.80–6.30	< 0.001
NEUT (%)	88.30 (82.73–91.98)	55.20 (51.95–58.30)	40–75	< 0.001
PLT (× 10^^9^/L)	173.20 ± 112.90	226.10 ± 65.82	125–350	0.104
CRP (mg/L)	157.70 (50.47–264.60)	ND	0–6	ND
PCT (ng/mL)	2.76 (0.55–19.64)	ND	0–0.046	ND
AST (μ/L)	28.00 (16.00–40.00)	19.00 (17.00–23.00)	M: (15–40) F: (13–35)	0.019
ALT (μ/L)	24.00 (15.00–36.00)	19.00 (14.00–27.00)	M: (9–50) F: (7–40)	0.253
ALP (μ/L)	81.00 (60.50–129.50)	ND	M: (45–125) F: (50–135)	ND
UA (μmol/L)	262.00 (193.00–340.00)	356.50 (304.80–396.00)	M: (208–428) F: (155–357)	0.009
CREA (μmol/L)	82.00 (62.50–119.00)	75.00 (58.50–80.50)	M: (59–104) F: (45–84)	0.064
CYSC (mg/L)	1.27 (1.10–1.90)	ND	0.40–1.10	ND
ALB (g/L)	32.18 ± 5.98	ND	40–55	ND
PA (mg/L)	102.00 (40.00–221.00)	ND	180–390	ND
Underlying diseases				
Diabetes	18 (42.86%)	ND	N/A	ND
Hypertension	16 (38.10%)	ND	N/A	ND
Chronic renal disease	5 (11.90%)	ND	N/A	ND
Solid cancer	9 (21.43%)	ND	N/A	ND
Hematological malignancy	5 (11.90%)	ND	N/A	ND
Pathogens				
*Escherichia spp.*	23 (54.76%)	ND	N/A	ND
*Klebsiella spp.*	3 (7.14%)	ND	N/A	ND
*Staphylococcus spp.*	7 (16.67%)	ND	N/A	ND
*Streptococcus spp.*	5 (11.90%)	ND	N/A	ND
Others	4 (9.52%)	ND	N/A	ND
Source of infection				
Primary bacteremia	13 (30.95%)	ND	N/A	ND
Urinary tract	9 (21.43%)	ND	N/A	ND
Intra-abdomina	6 (14.29%)	ND	N/A	ND
Skin and soft Tissue	4 (9.52%)	ND	N/A	ND
CVC	4 (9.52%)	ND	N/A	ND
Others	6 (14.29%)	ND	N/A	ND

P < 0.05 was considered statistically significant

Data are n (%), mean ± SD, and median (IQR)

*BSI* Bloodstream infection; *WBC* white blood cell; *NEUT* neutrophils; *PLT* platelet. *CRP* C-reactive protein; *PCT* procalcitonin; *AST* aspartate aminotransferase; *ALT* alanine aminotransferase; *ALP* alkaline phosphatase; *UA* uric Acid; *CREA* creatinine; *CYSC* cystatin C; *ALB*, albumin; *PA* prealbumin; *M* male; *F* female; *CVC* central venous catheter; *ND* Not determined; *N/A* not applicable

### Microbial Diversity in BSI and HCs

**Fig. 1:**
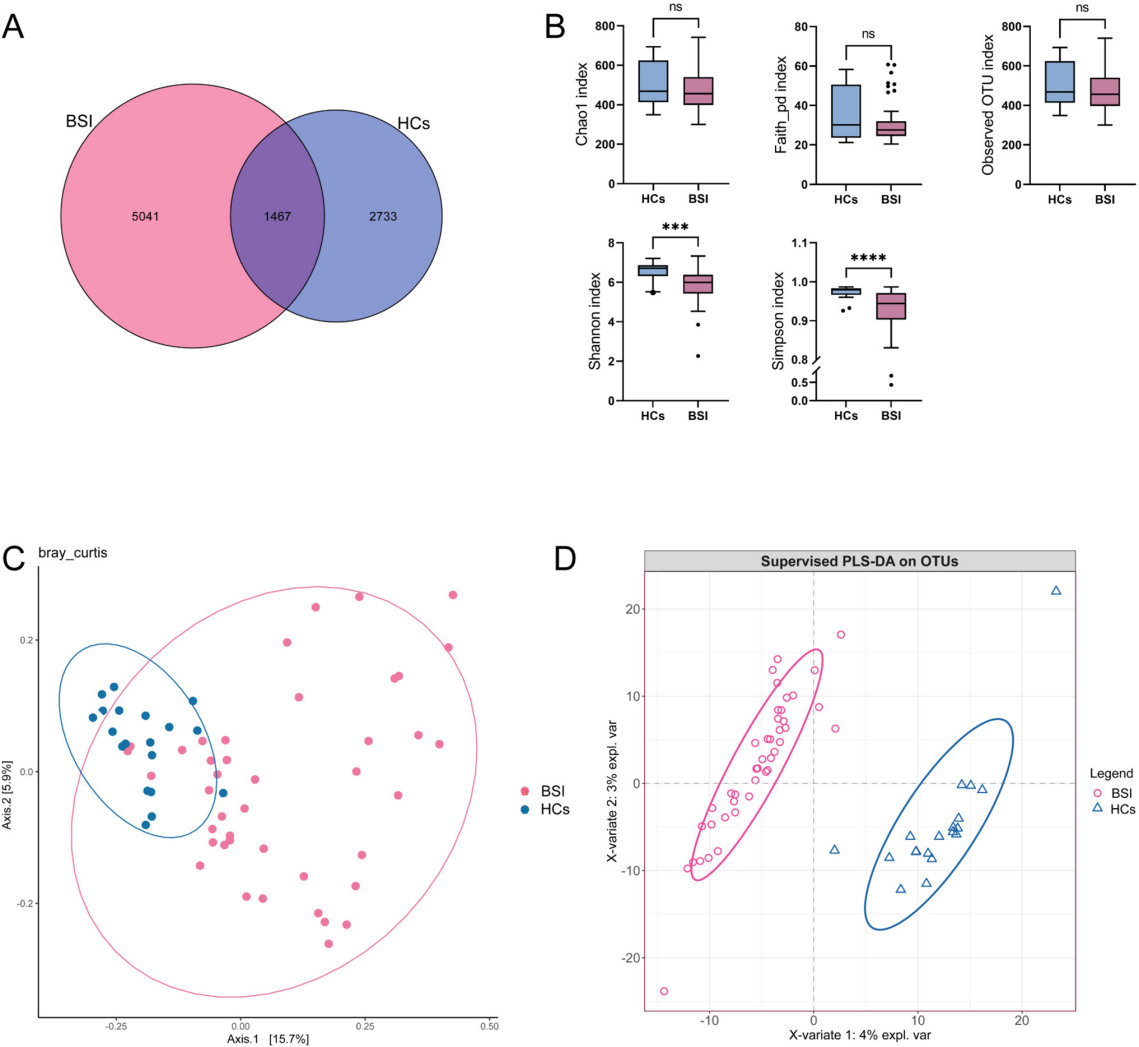
**A** Venn graph showing OTUs obtained in the BSI and HCs groups. **B** Boxplots representing the indices of Chao1, Faith pd, Observed OTUs, Shannon’s diversity, and Simpson’s diversity indices in the BSI and HCs groups. **C** Beta diversity calculated by PCoA of Bray–Curtis distances and PERMANOVA. The abscissa is a principal component, the ordinate is another principal component, and the percentage on the coordinate axis indicates the contribution of the two principal components to the sample difference. **D** PLS-DA was also introduced as a supervised model to show the microbiota variation among groups. PCoA, principal coordinate analysis; PERMANOVA, permutational multivariate analysis of variance analysis; HCs, healthy controls; PLS-DA, partial least squares discriminant analysis. ***p < 0.001, ****p < 0.0001. Non-significant comparisons are indicated by “ns”

There were 4,387,656 high-quality 16S rRNA-encoding sequences in the fecal samples from both groups. A total of 9241 OTUs were identified. Venn diagram analysis was conducted to clarify the characteristics of OTUs (Fig. 1A), and 1,467 OTUs were found to be common in two groups, while 2733 OTUs were found only in the HCs group, and 5041 OTUs in the BSI group.

Alpha diversity indices (Shannon and Simpson indices) showed a significant difference between the two groups (P < 0.001), with the BSI group having a notable decrease (Fig. 1B). PCoA analysis of beta diversity showed that the first principal component contributed 15.7% of the sample variance and the second principal component contributed 5.9% of the sample variance, with an overlap between the two groups, indicating some similarity in species composition between them (Fig. 1C). Beta diversity indices of the two groups were compared using the PERMANOVA method, which showed a significant difference in microbial compositional structure between the two groups (P = 0.001), indicating distinctive characteristics of bacterial species between the two groups. Furthermore, the intestinal flora in the HCs was relatively uniform among the different donors. PLS-DA analysis, a supervised analysis method that retains only information about differences between groups, was in general agreement with the results of PCoA, and the samples of the two groups were distinguished significantly, indicating that the structure of the intestinal flora differed significantly between the two groups (Fig. 1D).

### Comparison of Bacterial Spectra Between BSI Patients and HCs

**Fig. 2:**
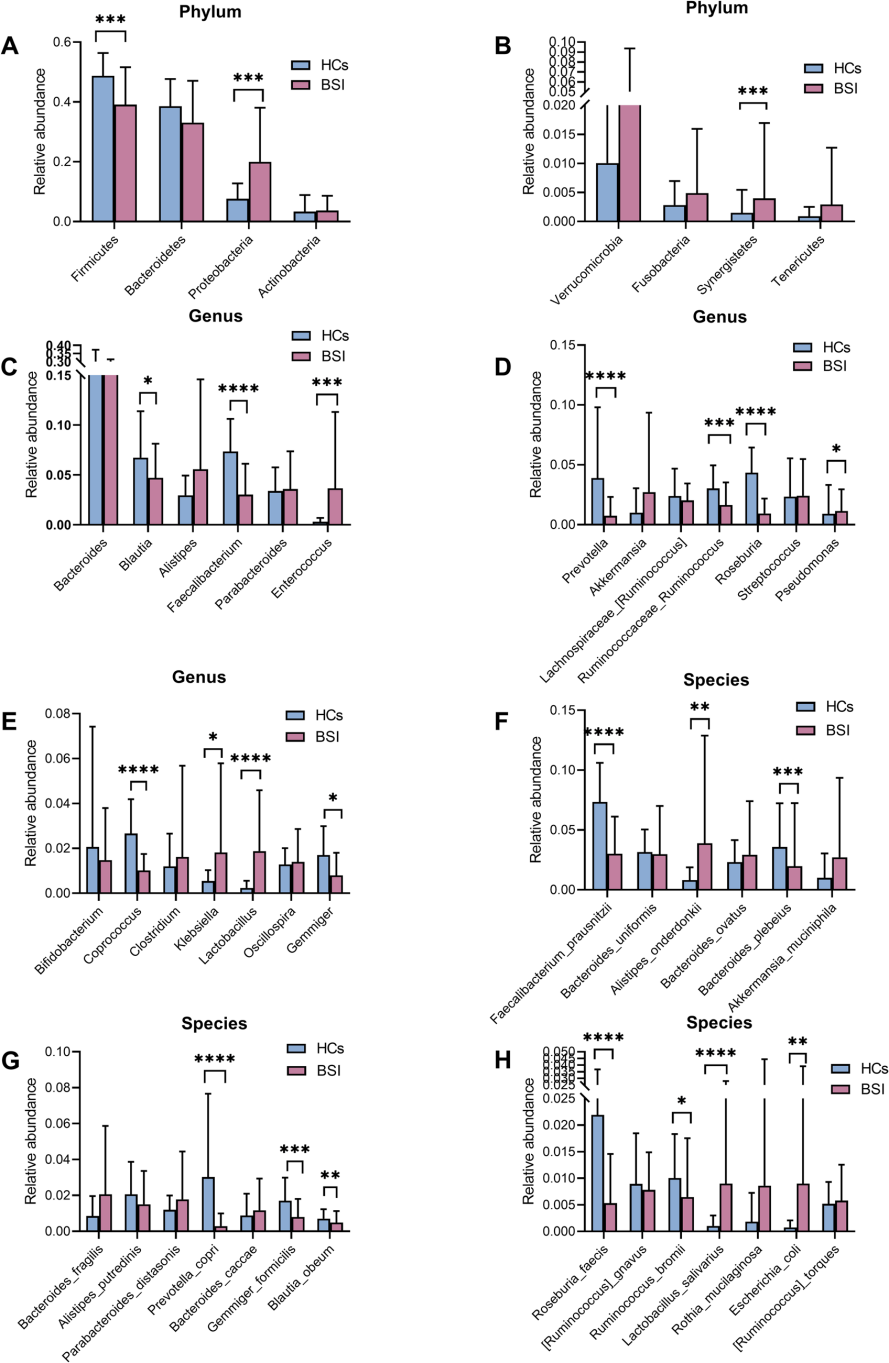
Histogram of relative abundance of intestinal flora in the BSI and HCs groups. **A**, **B** Comparison of the relative abundance of the top 8 bacteria at the phylum level between two groups. **C**, **D**, and** E** Comparison of the relative abundance of the top 20 bacteria at the genus level between two groups. **F**, **G**, and** H** Comparison of the relative abundance of the top 20 bacteria at the species level between two groups. *p < 0.05, **p < 0.01, ***p < 0.001, ****p < 0.0001

Cluster histograms were generated to show the bacterial features (see Supplementary Fig. 1). Phylum of Firmicutes, Bacteroidetes, and Proteobacteria dominated the eight phyla in the gut microbiota in both groups, but three phyla varied considerably between the two groups (Fig. 2A, B). Proteobacteria and Synergistetes were significantly more abundant, while Firmicutes were significantly less in the BSI than the HCs groups (P < 0.05). At the genus level, the abundances of *Blautia*, *Faecalibacterium*, *Ruminococcaceae_Ruminococcus*, *Roseburia*, *Prevotella*, *Coprococcus*, and *Gemmiger* were significantly lower, while that of *Enterococcus*, *Klebsiella*, *Pseudomonas* and *Lactobacillus* were significantly higher in the patients compared to that in the HCs (P < 0.05) (Fig. 2C, D, E). The top 10 bacterial species differed between the two groups. The abundances of *Faecalibacterium prausnitzii*, *Bacteroides plebeius*, *Prevotella copri*, *Gemmiger formicilis*, *Roseburia faecis*, *Blautia obeum*, and *Ruminococcus bromii* in the HCs exceeded those of the BSI group. However, the patients had significantly higher levels of *Alistipes onderdonkii*, *Lactobacillus salivarius*, and *Escherichia coli* than those in the HCs (P < 0.05) (Fig. 2F, G, H).

### Key Bacteria in BSI Patients and HCs

**Fig. 3:**
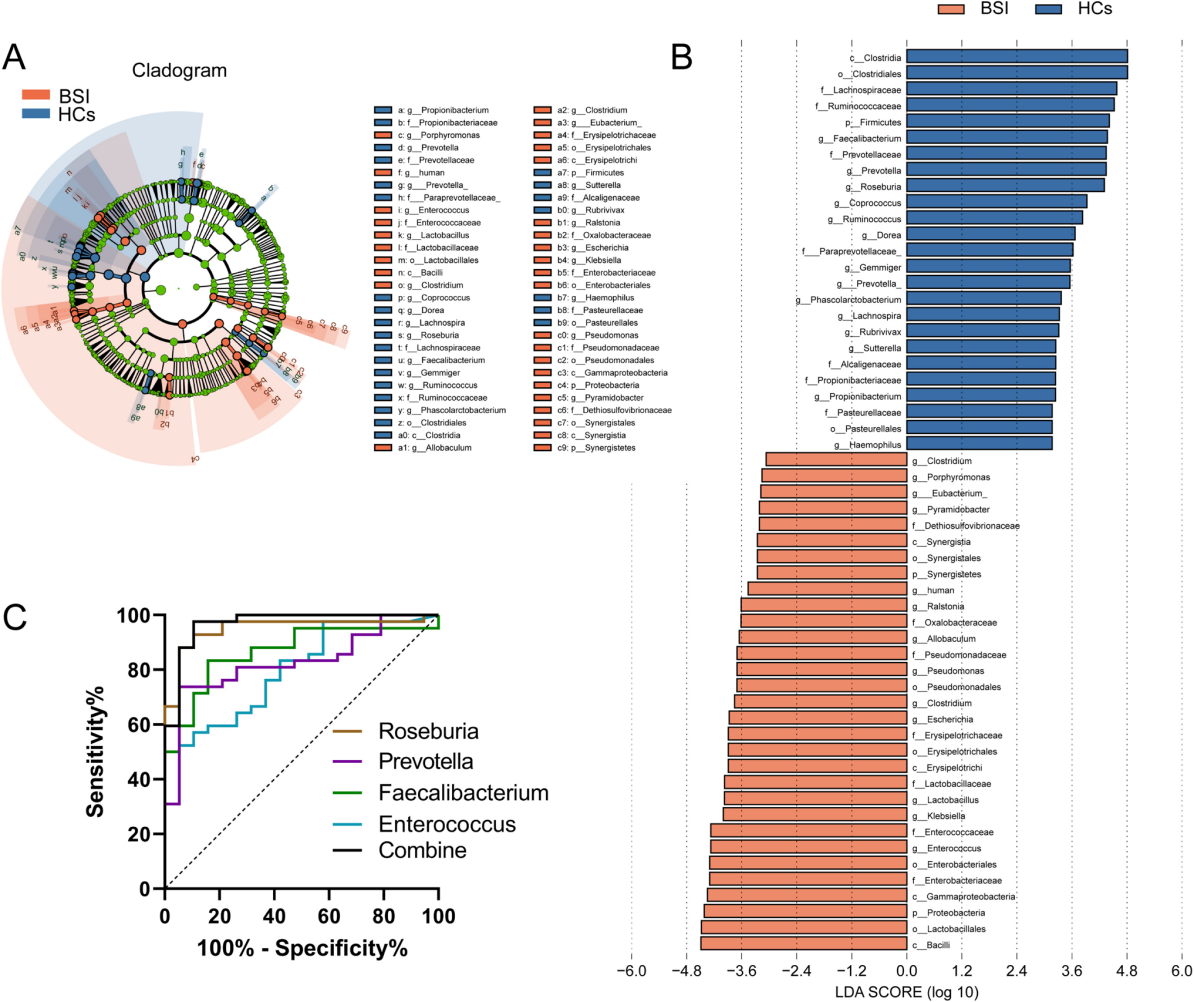
LEfSe analysis: the phylogenetic profiles of the specific bacterial taxa and predominant bacteria associated with BSI and HCs groups. **A** Cladogram:from the inside to the outside corresponds to different family classifications, and the line between the levels represents the relationship. The blue nodes represent the key species in the HCs group, while the orange nodes represent the key species in the BSI group. The green nodes represent normal species. The names of the species are on the right side of the figure. **B** The key species in each group were analyzed by LDA analysis. LDA score > 3.0; LEfSe, linear discriminant analysis effect size; LDA, linear discriminant analysis. **C** ROC curve analysis of the LDA > 4 genera of the BSI and HCs groups

Linear discriminant analyses (LDA) were performed to quantify the size of the major bacterial subpopulations between the two groups (Fig. 3A, B). As shown in the figure, significant differences across species were indicated by an LDA > 3. Compared to the standards, 31 bacterial species were detected in the patients and eight of them had an LDA > 4. On the other side, 25 bacterial species were detected in the HCs, nine of them with an LDA > 4. The strict (LDA > 4) version of LEfSe highlighted that, at the genus level, *Enterococcus* was enriched in the patients, whereas *Faecalibacterium*, *Prevotella*, and *Roseburia* were enriched in the HCs.

### Diagnostic Associations Between Gut *Bacteria* and Development of BSI

We further determine the associations between specific bacteria and BSI or HCs. The binary logistic regression models and ROC analyses were conducted to select the significant bacterial species (LDA > 4) at the genus level as the potential biomarkers for discriminating patients from HCs. There were four bacteria with an LDA > 4, including *Enterococcus* in the BSI group and *Faecalibacterium*, *Prevotella*, and *Roseburia* in the HCs. Based on the ROC curve, *Faecalibacterium*, *Prevotella*, and *Roseburia* showed good predictive potential (Fig. 3C). When the four bacteria were combined for diagnosis, the areas under the curve (AUC) reached 0.969 with a 95% CI of 0.924–1.000 (sensitivity = 97.62%, specificity = 89.47%, P < 0.0001) between BSI and HCs (see Supplementary Table 1). Our results demonstrate that fecal bacteria may become significant diagnostic markers and/or predictive potentials for BSI.

### Association Between Microbiota Composition and Clinical Parameters

**Fig. 4:**
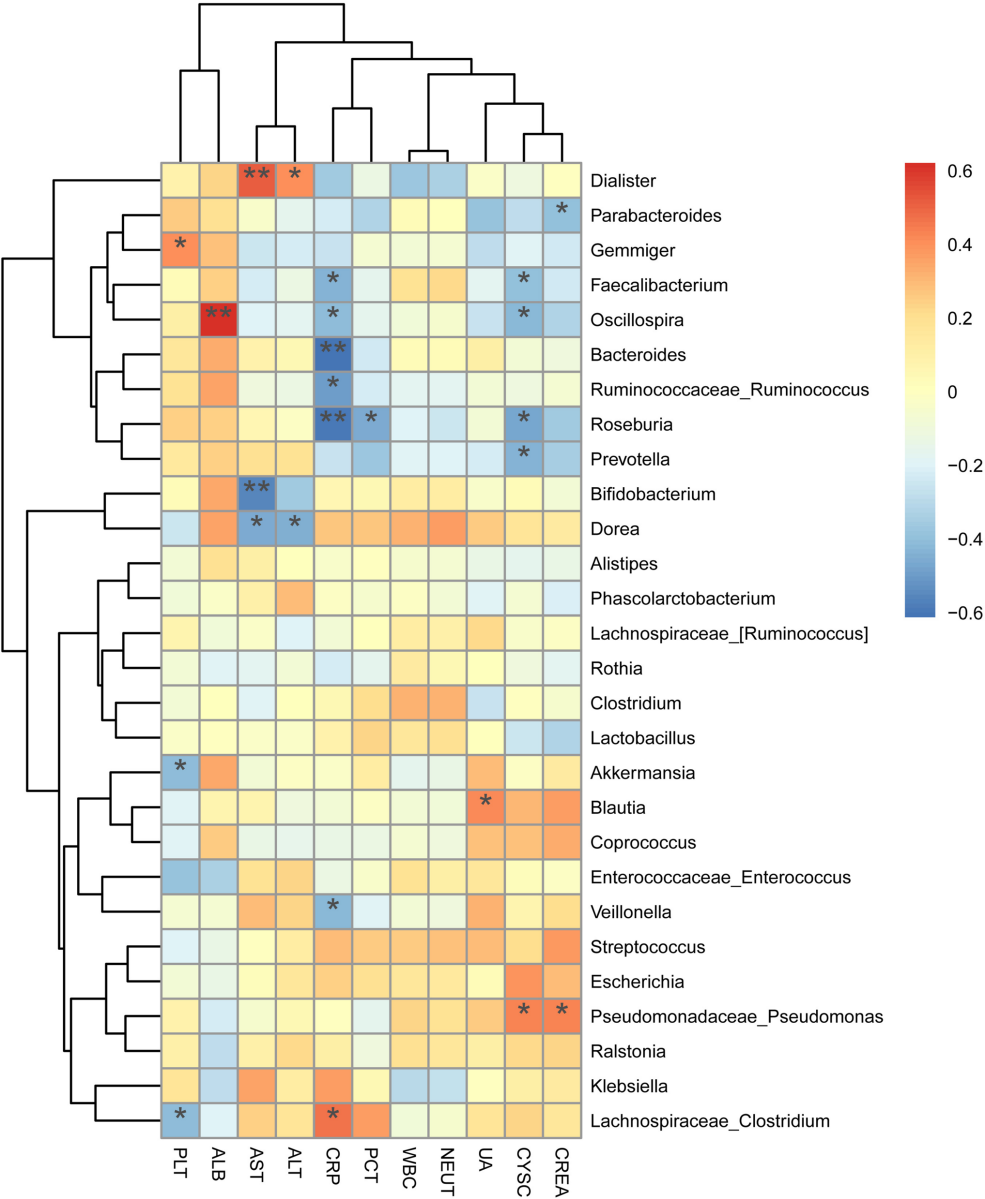
Heatmaps of interactions between species and environmental factors. Environmental factors are on the X-axis, and species are on the Y-axis; Blue represents negative correlation, and red represents positive correlation. *WBC* white blood cell; *NEUT* neutrophil; *PLT* platelet. *CRP,* C-reactive protein; *PCT* procalcitonin; *AST* aspartate aminotransferase; *ALT* alanine aminotransferase; *UA* uric Acid; *CREA* creatinine; *CYSC* cystatin C; *ALB* albumin; *p < 0.05, **p < 0.01

Relationships between the top 28 bacterial genera and clinical metrics were determined. The clinical metrics included measurements of WBC, neutrophil (NEUT), CRP, PCT, platelet (PLT), AST, alanine aminotransferase (ALT), UA, cystatin C (CYSC), albumin (ALB), and creatinine (CREA), etc. which reflected infectious status (Fig. 4). According to the findings, CRP was positively and significantly correlated with *Clostridium*, and negatively with *Veillonella*, *Roseburia*, *Ruminococcus*, *Bacteroides*, *Oscillospira*, and *Faecalibacterium* (P < 0.05). On the other hand, PCT and *Roseburia* were negatively and significantly correlated (P < 0.05). Interestingly, some of the gut bacteria were known to affect inflammatory pathways.

### Characteristics of the Gut Microbiota in BSI Patients with Various Pathogens

The patients were subclassified according to the pathogens isolated from their blood cultures: BSI-*Escherichia*, -*Klebsiella*, *-Staphylococcus*, and -*Streptococcus* groups. The Shannon and Simpson indices were lower in the *Escherichia* group, and the Simpson index was lower in the *Streptococcus* group (P < 0.05) among the patients compared to the HCs (P < 0.05). In addition, the dominant bacterial genera varied in each group (see Table S2), and that for the top 30 bacterial genera in each group are shown in Fig. S2.

## Discussion

Previous studies have revealed some mechanisms of sepsis development: increased permeability of the gut mucosa, tissue edema, decreased perfusion, dysregulation of tissue coagulation, shifts in the gut microbiome, apoptotic damage to the mucosal epithelia, and bacterial translocation (Bassetti et al., 2020). The resident intestinal microbiota has been stated to increase intestinal permeability (Cario et al., 2004), which would allow the outward transfer of germs to cause BSI. However, previous studies provide some data on patients with sepsis, whereas our study was conducted in patients with BSI, and this study will fill in the gap of the lack of characterization of the intestinal flora in patients with BSI.

Human intestinal homeostasis can be disrupted by factors including diet, stress, antibiotic and drug therapy, allergies, cancer, and related diseases, all of which make the host vulnerable to intestinal pathogens (Stecher, 2015). Whereas risk factors for developing BSI or sepsis include older age, immunosuppressive use, and potential co-morbidities (Hajj et al., 2018). In addition, risk factors for BSI in patients with hematologic disorders include the widespread use of empiric antimicrobial therapy, increasingly intensive chemotherapy regimens, and the frequent use of central venous catheters (Worth & Slavin, 2009). The factors affecting the intestinal flora are like the risk factors for BSI. That's why the exclusion criteria for our BSI group did not include diabetes, antibiotic use, radiotherapy, and cancer, just to try to evaluate the commonality of how these different factors affect the gut flora to the point of causing BSI.

Dysbiosis has been reported to increase sepsis hospitalizations by threefold (Prescott et al., 2015). In addition, low-diversity communities with limited resilience allowed pathogens to grow unrestrained (Kamada et al., 2013). Our investigation extended previous observations that BSI patients showed a loss of microbial richness, significantly lower alpha diversity, and increased pathogenic bacteria compared to HCs (Bassetti et al., 2020; Montassier et al., 2016). Furthermore, the beta diversity showed a significant difference in intestinal flora compositions between the patients and the HCs. The reduction of the symbiotic microbiota changed the overall dynamics between the microbiota and the host, resulting in low-grade inflammation, reduced anti-colonization ability, and altered susceptibility to infection (Forgie et al., 2019). In addition, our data demonstrate that the species composition in the HCs is quite different from that in the BSI patients, indicating that gut microbiota participate in the pathogenesis of BSI. In the PCoA plot, the cluster of BSI was bigger than the HCs group may be due to the BSI group containing patients with various diseases. Indeed, identifying common taxa within BSI can be more important for the robustness or easiness of diagnostics, therefore, studies should focus more on specific diseases to find more specific indicators.

Our LDA analyses identified certain bacteria of BSI that were different from those in HCs, which are supposed to be the main drivers of BSI. *Pseudomonas*, *Escherichia*, *Klebsiella*, and *Enterococcus* have been identified in our BSI patients and reported elsewhere (Stoma et al., 2021). On the other hand, our data indicate that *Faecalibacterium* were preferentially enriched in the HCs that are well-known anti-inflammatory microbes. Thus, their presence is often regarded as a sign of good gastrointestinal health (Gevers et al., 2014). Furthermore, *Faecalibacterium*, *Roseburia*, and *Coprococcus* have been shown to produce butyrate (Martin-Gallausiaux et al., 2021), a short-chain fatty acid (SCFA), which is beneficial to gut physiology, barrier function, and metabolism (Ziegler et al., 2003). Additionally, intestinal dysbiosis and extra-intestinal immunity are linked by the modulation of systemic immune responses by SCFA synthesis (Makki et al., 2018). SCFAs promoted the development of naive T cells into Th1 and Th17 cells and enhanced immunity if the host was engaged in a battle against pathogens (Park et al., 2015). Furthermore, physiological SCFA concentrations supported epithelial barrier function in the large intestine (Suzuki et al., 2008). For these reasons, our data support the notion that the reduction of SCFA-producing bacteria would cause susceptibility to BSI.

In BSI patients, *Faecalibacterium* and *Ruminococcus* levels were reduced, while *Escherichia coli* and *Klebsiella* were significantly higher than those in HCs. The high abundance of the latter two bacteria accounted for 61.9% of our patients and the bulk of BSI in diverse diseases. The long-held belief that these gut-derived organisms are supported by the close phylogenetic relationships between bacteria and among common enteric species, such as *Escherichia coli*, *Enterococcus faecium*, *Klebsiella pneumoniae*, and *Streptococcus mitis* (Tamburini et al., 2018). A sizable portion of the reported cases of BSI worldwide are caused by gram-negative bacterial species (Diekema et al., 2019), and they are the dominant *Enterobacteriaceae* in the gut microbiome.

Previous reports showed that 19 of the bloodstream isolates could be matched to bacteria in stool samples that were collected before sepsis (Graham et al., 2007). Among BSI patients who had allogeneic hematopoietic stem cell transplantation, the isolates were associated with intestinal domination, which was defined as a single bacterial taxon occupying at least 30% of the microbiota (Taur et al., 2012). Translocation from the intestinal microbial reservoir over a compromised gastrointestinal barrier into the bloodstream was a common cause of primary bloodstream infections with enteric organisms (See et al., 2017). Among our patients with *E. coli* dominance, approximately 34.78% of them had primary BSI, indicating that the gut was likely to be the source of the infections (Tamburini et al., 2018). In addition, the outward transfer of gut bacteria can be mediated via intravenous lines and locations where the integrity of the epidermal epithelium is damaged, allowing non-enteric commensal and environmental bacteria to enter the bloodstream (Tamburini et al., 2018).

Another major aim was to evaluate whether any of the identified bacteria could distinguish between HCs and patients who later developed BSI. According to the ROC analyses, *Prevotella*, *Faecalibacterium*, and *Roseburia* had high levels of sensitivity and specificity, with ROC-plot AUC values of 0.828, 0.867, and 0.951, respectively. In our findings, *Faecalibacterium, Prevotella*, and *Roseburia* were the dominant genera in HCs, and *Enterococcus* was the dominant genus in the patients. Indeed, an earlier research team developed a BSI risk index that can predict chemotherapy-related BSI based only on the pretreatment fecal microbiome (Montassier et al., 2016). In our results, the combined diagnostic value of four genera yielded a ROC-plot AUC value of 0.969, indicating that the four genera gained good diagnostic value. However, more specimens and maybe machine learning methods are needed to validate the combined diagnostic or predictive value of our observations. Probiotics have been extensively studied and have recently been used as a means of preventing sepsis in patients undergoing elective gastrointestinal surgery (Arumugam et al., 2016). Whether *Faecalibacterium*, *Prevotella*, and *Roseburia* can be used to prevent or treat BSI deserves further discussion.

Previous results show that CRP and PCT were the important indicators associated with a higher risk of BSI (Ratzinger et al., 2018). Our results show that CRP was negatively correlated with *Veillonella*, *Roseburia*, *Ruminococcus*, *Bacteroides*, *Oscillospira*, and *Faecalibacterium*; PCT was negatively correlated with *Roseburia.* Our observations provide further evidence that a diverse microbiome was associated with BSI. As mentioned above, *Roseburia*, *Ruminococcus*, and *Faecalibacterium* were related to the production of SCFAs and were negatively correlated with markers of inflammation. However, further investigation is also needed to find the specific connection.

In our study, alterations in intestinal flora in patients with BSI were quantitatively assessed, and the results show close relationships between BSI and gut microbiota disruption. As reported elsewhere, the microbiome predicts a patient’s propensity for disease, and its alteration averted the severity of the illnesses (Dickson, 2016). In conclusion, dysbiosis of fecal microbial ecology is a common condition present in patients with BSI. The proliferation or reduction of certain bacteria in the intestinal flora may be a risk factor for the development of BSI, and understanding the characteristics of the intestinal flora in BSI has some diagnostic or therapeutic value.

Our study has several limitations. First, patients with various underlying diseases were included in this study, which may have specific effects on gut microbiota. Addressing these confounding factors in the analysis would provide a clearer understanding of the relationship between gut dysbiosis and BSI. However, we could not fully explore how these conditions. Secondly, as a single-center study, its prediction of specific biological adverse states of BSI could be influenced by differences in diet and geographical locations. Our sample size is relatively small and the age of BSI patients is mostly over 50 years old, with limited representation. Finally, the study may have suffered from selection bias and bias due to variability in microbiome analysis. Therefore, our results need to be validated in a larger patient cohort, preferably in a multi-center study, to determine whether the results are replicable.

## Supplementary Information

Below is the link to the electronic supplementary material.

Supplementary file1 (PDF 496 KB)

Supplementary file2 (XLS 68 KB)

## Data Availability

The datasets presented in this study can be found in online repositories. The names of the repository(s) and accession number(s) can be found at: https://www.ncbi.nlm. nih.gov/, PRJNA932997.
