# Supplementary material for: Gut Microbiota Dysbiosis Facilitates Susceptibility to Bloodstream Infection
Source: J Microbiol. 2024 Dec 2;62(12):1113–24. doi: 10.1007/s12275-024-00190-5 (PMC11652583; doi:10.1007/s12275-024-00190-5)
Supplement: Supplementary file 1 — Supplementary file1 (PDF 496 KB) [file 12275_2024_190_MOESM1_ESM.pdf]

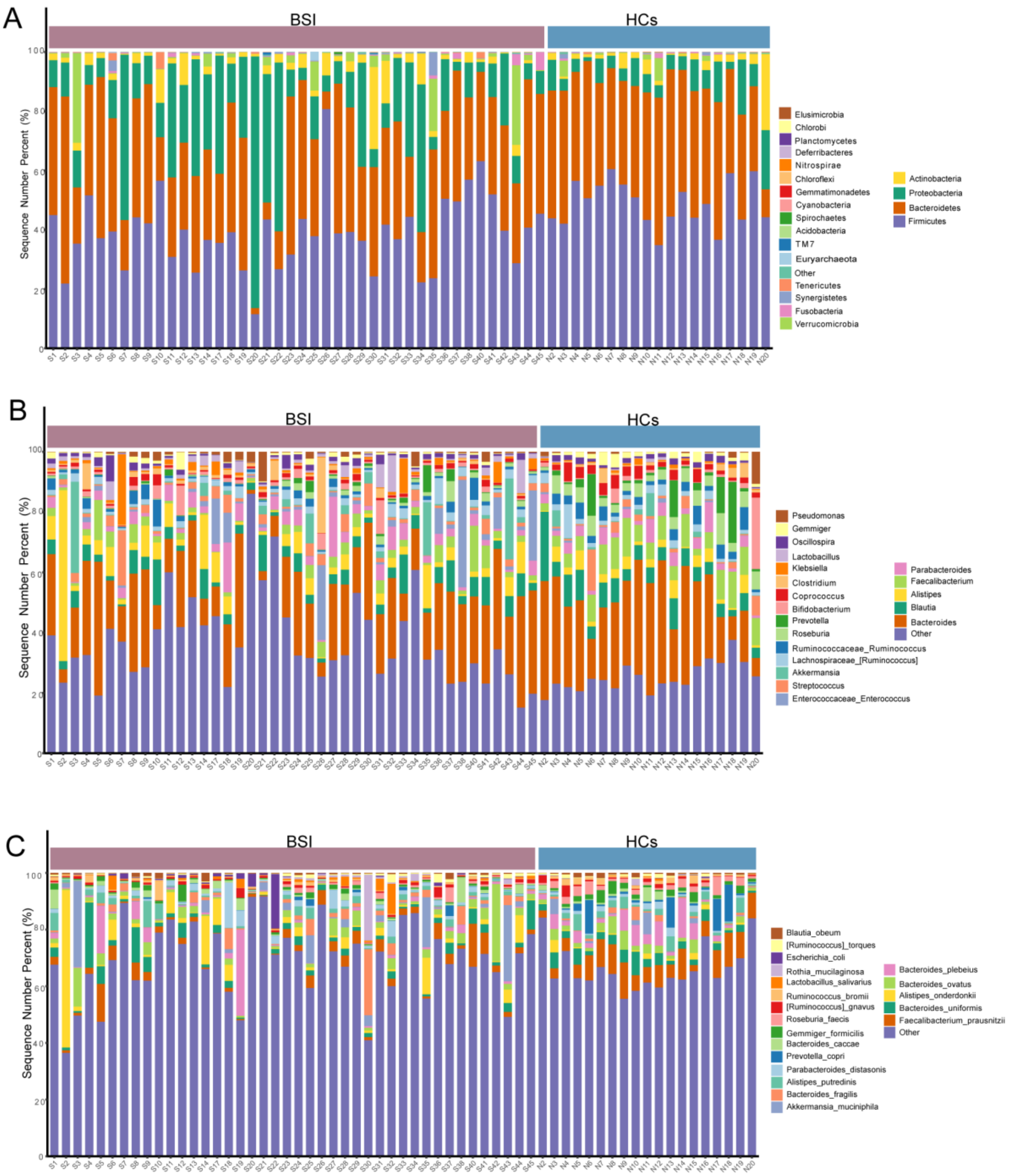

Fig. S1. Analysis of relative gut microbiota abundance in the BSI and HCs. Each column corresponds to one sample. **A** Relative abundance of the gut microbiota at the phylum level. **B** Relative abundance of the gut microbiota at the genus level. **C** Relative abundance of the gut microbiota at the species level. "Other" represents the remaining bacteria. HCs, healthy controls;

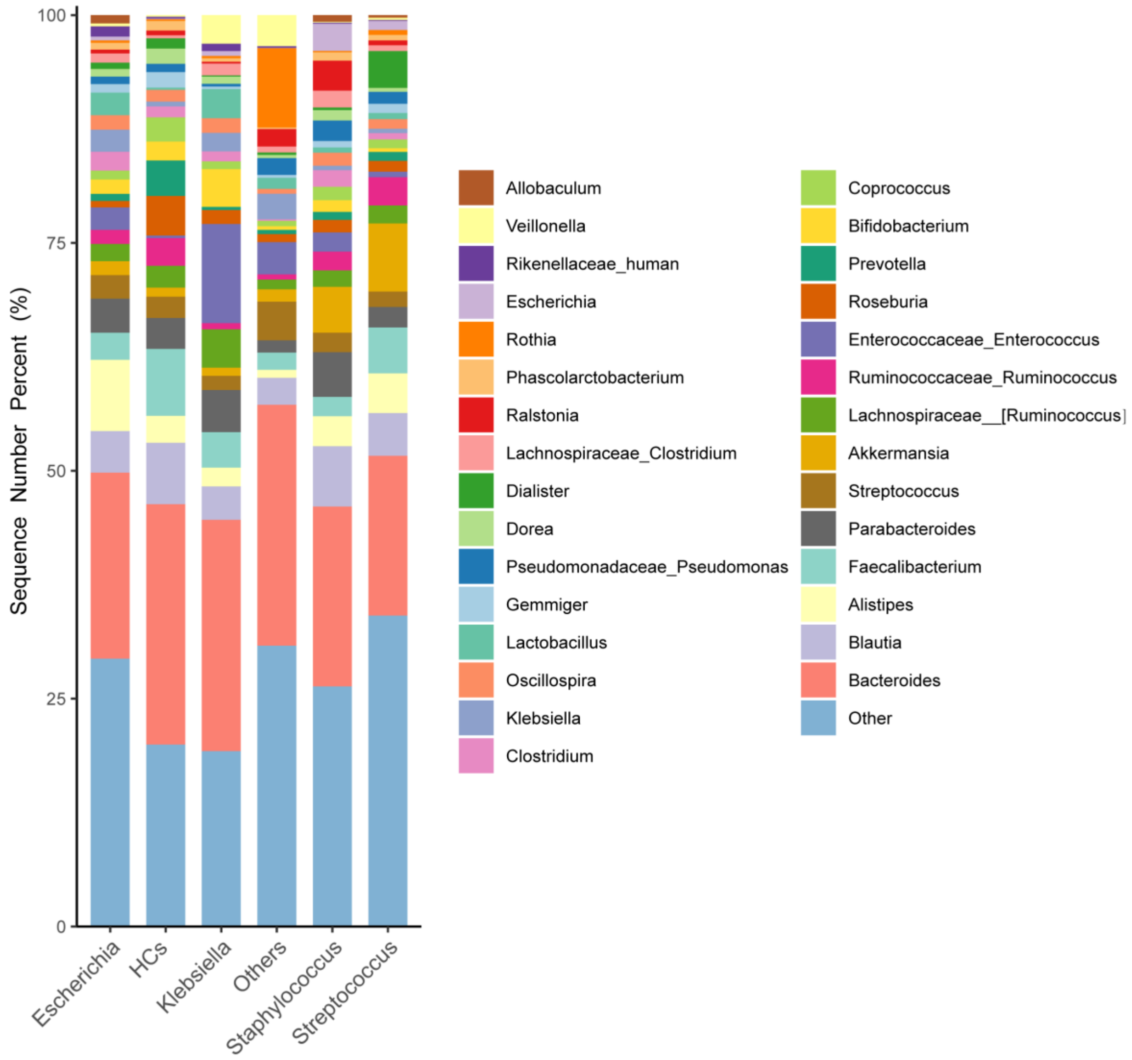

Fig. S2. Relative abundances of the gut microbiota at the genus level. Each column corresponds to the mean of the total samples in each group. “Other” represents the remaining bacteria. HCs, healthy controls;

Table S1. Performance of the LDA > 4 genera in the diagnosis of BSI

	Faecalibacterium	*Prevotella*	*Roseburia*	*Enterococcus*	Combine
AUC	0.867	0.828	0.951	0.791	0.969
95% CI	0.775-0.960	0.723- 0.934	0.895-1.000	0.674- 0.907	0.924-1.000
Cut-off value	0.0455	0.005316	0.01942	0.008299	N/A
Sensitivity (%)	83.33	73.81	88.10	52.38	97.62
Specificity (%)	84.21	94.74	94.74	94.74	89.47
P-value	< 0.0001	< 0.0001	< 0.0001	0.0003	< 0.0001

Notes: P < 0.05 was considered statistically significant; AUC, areas under curve; N/A, not applicable;

Table S2. Characteristics of intestinal flora in BSI patients induced by different pathogens

Pathogen of BSI	*Escherichia spp.* (n=23)	*Klebsiella spp.* (n=3)	*Staphylococcus* spp.(n=7)	*Streptococcus* spp.(n=5)	HCs (n=19)
Source of infection					
Primary bacteremia	8	2	0	1	N/A
Urinary tract	9	0	0	0	N/A
Intra-abdomina	5	1	0	0	N/A
Skin and soft Tissue	0	0	2	2	N/A
CVC	0	0	4	0	N/A
Others	1	0	1	2	N/A
Alpha diversity					
Shanon index	6.05（5.43-6.32）[a]	6.28（5.84-6.82）	6.32（4.86-7.00）	6.42（4.09-6.72）	6.71（6.31-6.86）
Simpson index	0.94（0.90-0.97）[a]	0.97（0.96-0.98）	0.97（0.88-0.98）	0.97（0.67-0.97）[b]	0.98（0.96-0.98）
Gut bacteria (Top 5 genus)	*Bacteroides*	*Bacteroides*	*Bacteroides*	*Bacteroides*	*Bacteroides*
	Alistipes	*Enterococcus*	*Blautia*	*Akkermansia*	*Faecalibacterium*
	Blautia	*Parabacteroides*	*Akkermansia*	*Faecalibacterium*	*Blautia*
	Parabacteroides	*[Ruminococcus]*	*Parabacteroides*	*Blautia*	*Roseburia*
	Faecalibacterium	*Bifidobacterium*	*Ralstonia*	*Alistipes*	*Prevotella*

Notes: [a]: compared with HCs P < 0.001 [b]: compared with HCs P < 0.05; CVC, central venous catheter; HCs, healthy controls; N/A, not applicable;
